# Supplementary material for: Safety and Evaluation of the Immune Response of Coronavirus Nosode (BiosimCovex) in Healthy Volunteers: A Preliminary Study Extending the Homeopathic Pathogenetic Trial
Source: Medicines (Basel). 2022 Dec 30;10(1):8. doi: 10.3390/medicines10010008 (PMC9865918; doi:10.3390/medicines10010008)
Supplement: Supplementary file 1 [file medicines-10-00008-s001.zip › medicines-2123686-supplementary.pdf]

Supplementary Tables of individual subject changes after treatment measuring IL-6, Absolute lymphocyte count, CD4, and CD8

Table S1: IL- 6 (0.00-7.00 pg/mL)Individual subject changes after treatment in comparison with baseline, (RM-ANOVA test)

Subject	Baseline	Day 17	Day 34	Day 60
1	1.50	3.67	10.4	3.34
2	3.02	5.49	9.22	7.58
3	2.00	6.42	44.6	5.93
4	1.5	2.65	24.5	1.5
5	2.5	8.23	41.8	1.59
6	3.7	8.77	38.1	1.5
7	1.5	3.91	23.4	4.14
8	1.5	12.0	39.5	24.26
9	1.5	4.9	35.7	4.5
10	1.5	4.73	23.1	3.95
Mean	2.022	6.077	29.032	5.829
Note: p-value 0, calculated using the RM-ANOVA test				

Table S2: Individual subject CD panel in individuals during the study

(t-Test, Paired two Sample for Means)

Subject	Absolute Lymphocyte count (990.00 - 3150.00)/ul		Absolute CD4 (424.00-1509.00)/ul		Absolute CD8 (169.00-955.00)/ul	
	Day 34	Day 60	Day 34	Day 60	Day 34	Day 60
1.	2605	2428	630	567	1046	1153
2.	1563	1699	505	526	563	699
3.	2700	2666	1453	1520	427	359
4.	2281	2690	1037	1244	487	538
5.	1570	1809	722	826	300	351
6.	1754	1733	801	846	426	444
7.	1576	1875	628	693	527	585
8.	2854	3004	1010	1179	803	848
9.	2445	2097	633	614	908	751
10.	2639	2878	916	993	1036	1149
Mean	2198.7	2287.9	833.5	900.8	652.3	687.7
P value	0.25587		0.026799		0.2373	
